# Quantification of morphochemical changes during *in situ* enzymatic hydrolysis of individual biomass particles based on autofluorescence imaging

**DOI:** 10.1002/bip.23347

**Published:** 2019-12-23

**Authors:** Dimitrios Kapsokalyvas, Joachim Loos, Ilco A. L. A. Boogers, Maaike M. Appeldoorn, Mirjam A. Kabel, Marc Van Zandvoort

**Affiliations:** ^1^ Department of Molecular Cell Biology, GROW CARIM, Maastricht University Maastricht ER The Netherlands; ^2^ Materials Science Center, Royal DSM N.V. Geleen The Netherlands; ^3^ Biotechnology Center, Royal DSM N.V. Delft The Netherlands; ^4^ Laboratory of Food Chemistry Wageningen University Wageningen The Netherlands; ^5^ Department of Molecular Cell Biology, CARIM, GROW, MHeNs, NUTRIM Maastricht University Maastricht ER The Netherlands; ^6^ Institute for Molecular Cardiovascular Research (IMCAR) RWTH Aachen University Aachen Germany

**Keywords:** biomass, cellulose, enzymatic hydrolysis, FLIM, pretreated corn stover (pCS), two‐photon microscopy

## Abstract

Enzymatic hydrolysis of biomass is an established method for producing biofuels. Lignocellulosic biomass such as corn stover is very inhomogeneous material with big variation on conversion rates between individual particles therefore leading to variable recalcitrance results. In this study, we used noninvasive optical microscopy techniques, such as two‐photon microscopy and fluorescence lifetime imaging microscopy, to visualize and analyze morphological and chemical changes of individual corn stover particles pretreated with sulfuric acid during hydrolysis. Morphochemical changes were interpreted based on the fluorescence properties of isolated building blocks of plant cell wall, such as cellulose, hemicellulose, and lignin. Enzymatic hydrolysis resulted in particle size reduction, side wall collapse, decrease of second harmonic signal from cellulose, redshifting of autofluorescence emission, and lifetime decrease attributed to the relative increase of lignin. Based on these observations, tracking compositional change after hydrolysis of individual particles was accomplished. The methodologies developed offer a paradigm for imaging and analyzing enzymatic hydrolysis *in vitro* and *in situ*, which could be used for screening enzymes cocktails targeting specific recalcitrant structures or investigating locally enzyme anti‐inhibitory agents.

## Graphical abstract

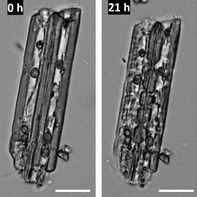

**Abbreviations**
FLIM: fluorescence lifetime imaging microscopy
OSX: oat spelt xylan
pCS: pretreated corn stover
SHG: second harmonic generation
TPM: two‐photon microscopy
WAX: wheat arabinoxylan

## 1 INTRODUCTION

Research on the use of lignocellulosic plant biomass for the production of bioethanol or chemicals has been a very active field during the last decades.^1^ The so‐called *second‐generation biofuels* are produced from residue biomass such as corn stover, sugar cane bagasse, or woody biomass. Biomass is mainly composed of varying ratios of cellulose, hemicellulosic xylan, and lignin, depending on the species. These molecules, through interactions between cellulose and hemicellulose, and covalent linkages between hemicellulose and lignin, form the plant cell wall, which is impermeable to the external environment.^2^ Because of this tight interconnection, release of monosaccharides (which can be converted to biofuels) from plant biomass is difficult.^1^ In industrial scale production, usually a combined process of pretreatment, enzymatic hydrolysis, and fermentation is applied.

Several pretreatment methods, such as dilute acid, hot water, or biological agents, have been developed. They offer variable outcomes depending on biomass type and treatment parameters.^3^ In general, during acid catalyzed hydrothermal pretreatment (partial) delignification or (partial) solubilization of the hemicellulosic fraction occurs in addition to a (partial) decrease of cellulose crystallinity.^4^, ^5^, ^6^ Hereby, the plant cell wall material is opened up, and enzymes have increased access to the polysaccharides to release the fermentable sugars aimed at.^7^ Both pretreatment and enzymatic hydrolysis are important for the overall conversion of polysaccharides to monosaccharides.^5^, ^8^ In the field of enzyme hydrolysis, the main effort is on developing more efficient enzyme cocktails to increase glucose and xylose conversion, and simultaneously offer viable solutions for industrial production.^9^, ^10^

Chemical characterization of pretreatment and hydrolysis is commonly performed with various analytical techniques, such as high‐pressure liquid chromatography (HPLC),^11^, ^12^ nuclear magnetic resonance (NMR),^13^, ^14^ and near infrared spectroscopy (NIRS).^15^, ^16^ All these techniques can provide detailed information on the overall chemical composition of the sample, leading to an accurate calculation of the glucose and xylose conversion efficiency, which is critical information for further optimization.

Hydrolysis induces great morphological and chemical changes on biomass particles. Cellulose conversion to glucose is eliminating a fundamental structural element of the plant cell wall, which leads to its collapse. Additionally, since biomass is an inhomogeneous material, these changes have variable effects on individual particles which leads to different hydrolysis rates for different parts of the plant.^17^ Local morphology and treatment‐induced morphological changes can affect the access of enzymes and the overall conversion efficiency.

To gain insight into the mechanisms of these processes also microscopic techniques have been used. Electron microscopy has been applied for detailed analysis of the hydrolytic mechanisms of cellulases^18^ as well as the effect of pretreatment on biomass.^18^, ^19^, ^20^, ^21^ Such techniques can provide morphological information with very high resolution on individual biomass particles. Optical microscopy techniques, which are less invasive, have been used to study cellulose activity^22^, ^23^, ^24^ as well as the effect of pretreatment.^25^, ^26^ Since biomass particles can be observed in their native environment with minimal sample preparation, the effect of enzymes can be studied locally on individual particles without the need of indirect quantification procedures.

In this study, we applied two‐photon microscopy (TPM) for imaging individual particles of pretreated corn stover (pCS). TPM is an optical microscopic technique with good penetration depth and tomographic capabilities, providing full three‐dimensional images. Additionally, it has the unique capability to image cellulose based on its SHG signal, without the requirement of staining.^27^ Combined with the autofluorescence properties of biomass, imaging the morphology of biomass particles is possible without the need for staining, or any additional sample preparation. This further enables imaging of biomass *in situ* during hydrolysis. Finally, TPM when combined with fluorescence lifetime imaging microscopy (FLIM) can provide chemical information of the sample. TPM has been used for imaging lignocellulosic samples, such as sugar cane bagasse samples,^25^, ^28^ soft wood,^29^ and poplar and wheat straw,^30^ in order to investigate the morphological and chemical changes induced by different pretreatments, and enzymatic hydrolysis. Hereto, the autofluorescence properties of the plant cell wall polymers were first investigated and afterward used to interpret the autofluorescence signal of pCS samples. To fully exploit the advantages of the noninvasive nature of optical microscopy, *in situ* imaging of hydrolysis was performed to image the morphological and chemical changes induced by hydrolytic enzymes on individual biomass particles. Microscopic evidence of enzymatic activity on individual structures could offer an alternative screening method for enzyme cocktails, which could more efficiently target recalcitrant structures or identify anti‐inhibitory factors which could optimize the conversion process.

## 2 MATERIALS AND METHODS

### 2.1 Samples

#### 2.1.1 Cell wall components

Autofluorescence properties of the isolated plant cell wall components, − cellulose, hemicellulose, and lignin − were investigated. Since these components cannot be found isolated in nature, synthesized or extracted samples were used for this part of this study.

##### Cellulose

Cellulose is a polysaccharide and an important structural component of the cell wall of plants. It consists of crystalline and amorphous regions. We investigated the autofluorescence properties of both high crystalline and amorphous celluloses. Four reference samples were used, the high crystallinity celluloses (a) Avicel (11 365, Sigma‐Aldrich), (b) α‐cellulose (C8002, Sigma‐Aldrich) and the amorphous celluloses, (c) D4MRes, and (d) regenerated amorphous cellulose (RAC). Avicel is a microcrystalline cellulose powder extracted from wood. α‐Cellulose is the carbohydrate portion of a cellulosic material that is not dissolved in a 17.5% solution of sodium hydroxide at 20 °C, usually extracted from cotton or wood. The D4MRes amorphous cellulose is extracted from empty fruit bunches from oil palms as described elsewhere^31^ and involved the removal of lignin with peracetic acid followed by an alkaline removal of hemicelluloses. RAC (prepared as described elsewhere^32^) is a phosphoric acid treated Avicel cellulose, a treatment which has shown to reduce crystallinity content of crystalline cellulose, depending on the conditions, up to 80%.^33^

##### Hemicelluloses

Hemicellulose describes a family of matrix polysaccharides including xylan, glucuronoxylan, arabinoxylan, glucomannan, and xyloglucan. In contrast to cellulose, hemicellulose has a heterogeneous, amorphous structure. Hemicelluloses from three different sources were analyzed: (a) wheat arabinoxylan (medium viscosity, Megazyme, Wicklow, Ireland, xylose: 59%, arabinose: 36%), (b) Birch xylan (Magazyme, Wicklow, Ireland, xylose: 86%, other sugars: 5%), and (c) oat spelt xylan (Sigma‐Aldrich, Steinheim, Germany, xylose: ≥70%, arabinose: 10%, glucose: 15%).

##### Lignin

Lignin is an organic polymer important for the formation of cell wall. Three different samples were investigated: (a) Alcell organosolv lignin (mixed hardwoods: maple, birch, and poplar, Repap Technology, Canada), (b) Kraft lignin Indulin AT (softwood, Meadwestvaco, USA), and (c) soda lignin Protobind 1000 (wheat straw/Sarkanda grass, GreenValue S.A., Switzerland). According to chemical composition analysis of these lignin samples,^34^ the acid insoluble lignin content was 94 w/w% for Alcell, 90 w/w% for Indulin AT, and 85 w/w% for Protobind 1000, while acid soluble lignin was 2 w/w%, 2 w/w%, and 5 w/w% correspondingly.

##### Composite biomass sample

In order to create a biomass‐like sample, for validation purposes, a mixture of α‐cellulose, Birch xylan, and Protobind 1000 lignin were mixed in water with a ratio of 4:3:3, which is similar to the ratio that these components can be naturally found in corn stover.^35^

#### 2.1.2 Pretreated corn stover samples

Corn stover samples were collected and treated based on NREL guidelines. Pretreatment was performed with 0.6 w/w% H_2_SO_4_ at 190 °C for 1 minute holding time. Samples were stored at 4 °C until further analysis. This sample was used for hydrolysis monitoring.

### 2.2 Imaging and analysis

A Leica TCS SP5 (Leica Microsystems GmbH, Wetzlar, Germany), two‐photon microscope was used for imaging and measurement of fluorescence spectra and fluorescence decay of samples. The excitation source was a Ti:Sapphire Chameleon Ultra II (Coherent Inc., Santa Clara, California) laser, at 820 nm. A Leica objective HCX APO L 20x/1.00 W was used for excitation and epicollection. Laser power range was 40 to 80 mW. The laser beam is elliptically polarized. Three types of measurements were performed: (a) spectral, (b) intensity (called TPM in Section 3), and (c) fluorescence lifetime (called FLIM in Section 3). The spectrum of a fluorophore depends on the de‐excitation transitions and the distance of the excited from to the ground electronic state, while lifetime depends on the rate that these transitions take place. All cell wall components samples were diluted in distilled water at 1 μg/ml and mounted on a microscope glass covered with a coverslip for imaging.

#### 2.2.1 Spectral

**Figure 1:**
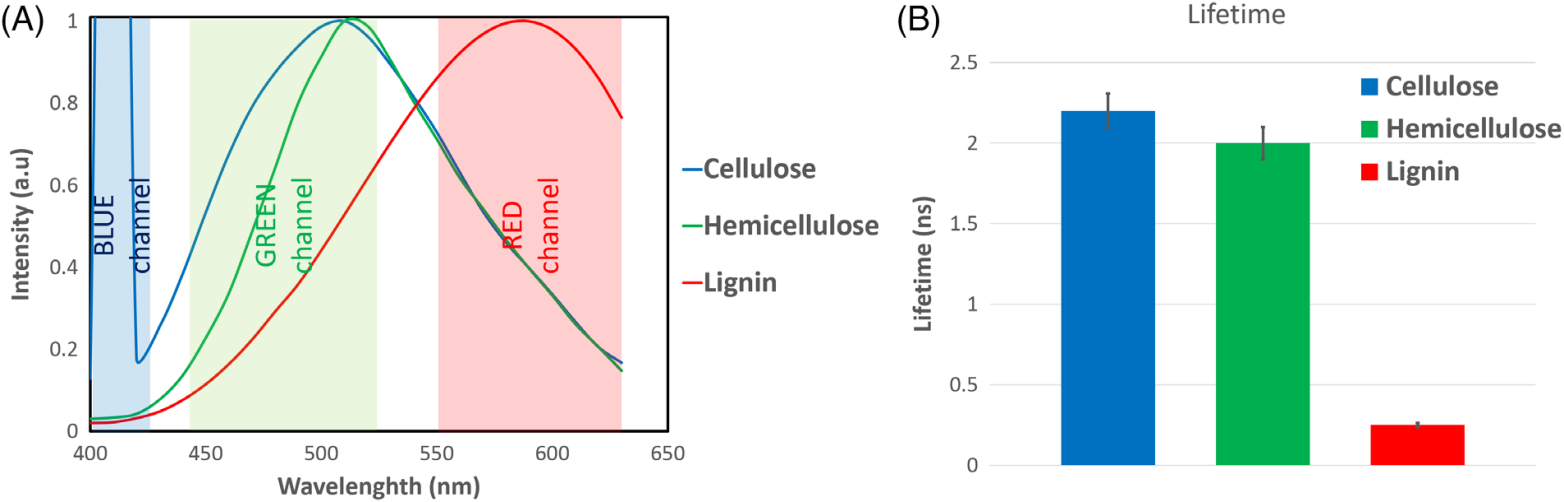
Autofluorescence properties of plant cell wall building blocks. A, Autofluorescence spectra of cellulose and xylan have a major peak at 510 and 520 nm, while lignin at 580 nm. Cellulose has also (SHG) signal at 410 nm, when excited at 820 nm. In the background of the graph the detection bandwidth used for subsequent TPM imaging of pCS is indicated. B, Autofluorescence lifetime of cellulose (2.16 ns) and hemicellulose (2.01 ns) are similar and significantly higher than that of lignin (0.24 ns)

Emission spectra were acquired with the wavelength scanning mode of the microscope. Totally, 24 images at 10 nm intervals from 400 to 640 nm were acquired, combined to a multispectral stack and were analyzed with Fiji (ImageJ).^36^ For each multispectral stack, a region of interest, containing the sample, was selected, and the spectrum was measured. Spectra presented Figure 1 and in Supporting Information Figures S1M, S2J, and, S3J are average normalized spectra from six multispectral images in each case. In Figure S1M, SHG values are normalized on the peak of the autofluorescence emission at 510 nm.

#### 2.2.2 Two‐photon microscopy

For imaging, two descanned PMT (photomultiplier tube) detectors were used for autofluorescence detection. One detector was set at 440 to 525 nm (green channel), where mainly cellulose and xylan emit, and the other was set at 550 to 630 nm (red channel) where mainly lignin emits. One additional detector in the forward direction was used to detect SHG (blue channel) from cellulose. A laser cutoff filter (SP 680) and a band pass filter (FF01‐400/40, Semrock Inc., Rochester, New York) were mounted before the forward detector. Imaging in three spectral channels was chosen, instead of spectral imaging, for increasing the acquisition speed and reducing the possibility of photodamage.

#### 2.2.3 Fluorescence lifetime imaging microscopy

FLIM is a technique that enables the measurement of the fluorescence decay of fluorophores, with high spatial and temporal resolution.^37^ FLIM measurements were performed with a Becker & Hickl TCSPC (Time Correlated Single Photon Counting) module (SPC 830, Becker & Hickl GmbH, Berlin, Germany) adapted on the Leica TCS SP5. Visualization of the acquired images was performed with the dedicated software SPCM9.5 (Becker & Hickl GmbH, Berlin, Germany). Detection was performed at 440 to 640 nm bandwidth. Acquisition was performed with 256‐pixel resolution and 256 ADC (analog to digital converter) resolution. Two‐exponential fitting, deconvolution, and fluorescence decay analysis were performed with the dedicated software SPC‐Image 3.5 (Becker & Hickl GmbH, Berlin, Germany). The *incomplete multiexponentials* fitting model was used to account for the small period between each excitation pulse (12.5 ns). Mean lifetime was calculated as *τ*_mean_ = (*α*_1_ ∙ *τ*_1_ + *α*_2_ ∙ *τ*_2_)/(*α*_1_ + *α*_2_) where *τ*_1_ and *τ*_2_ are the short and long lifetimes and *α*_1_ and *α*_2_ are their corresponding amplitudes.

#### 2.2.4 Fluorescence ratio

Fluorescence ratio (FR) was calculated by dividing the red channel over the green channel, pixel by pixel. Before division images were processed with a low and a high threshold to remove noise and saturated pixels. The acquired images were 8‐bit with intensity levels between 0 and 255. The threshold levels used for all images were *low threshold*: 15, *high threshold*: 250. FR values are presented as mean ± SD.

#### 2.2.5 Enzymatic hydrolysis monitoring

The two‐photon microscope was used for monitoring hydrolysis of individual particles. To perform this measurement, a custom windowed chamber was developed. The pCS sample was diluted in a 0.9 ml of 0.2 M acetate buffer at pH 5 and an appropriate amount of hydrolytic enzyme mixture containing cellulases and hemicellulases (DSM, Delft, Netherlands) was added. The sample was placed inside the chamber and under the microscope on a heating plate (50 °C) and was monitored for 18 hours. This time period is enough to convert most of the cellulose to sugars. Brightfield, TPM, and FLIM images were acquired before and after 18 hours of hydrolysis. Since the objective is water immersion, in order to avoid evaporation during imaging, a mineral oil‐based medium (Immersol W 2010, Zeiss, Germany) with refractive index similar to water was used instead.

## 3 RESULTS AND DISCUSSION

**Figure 2:**
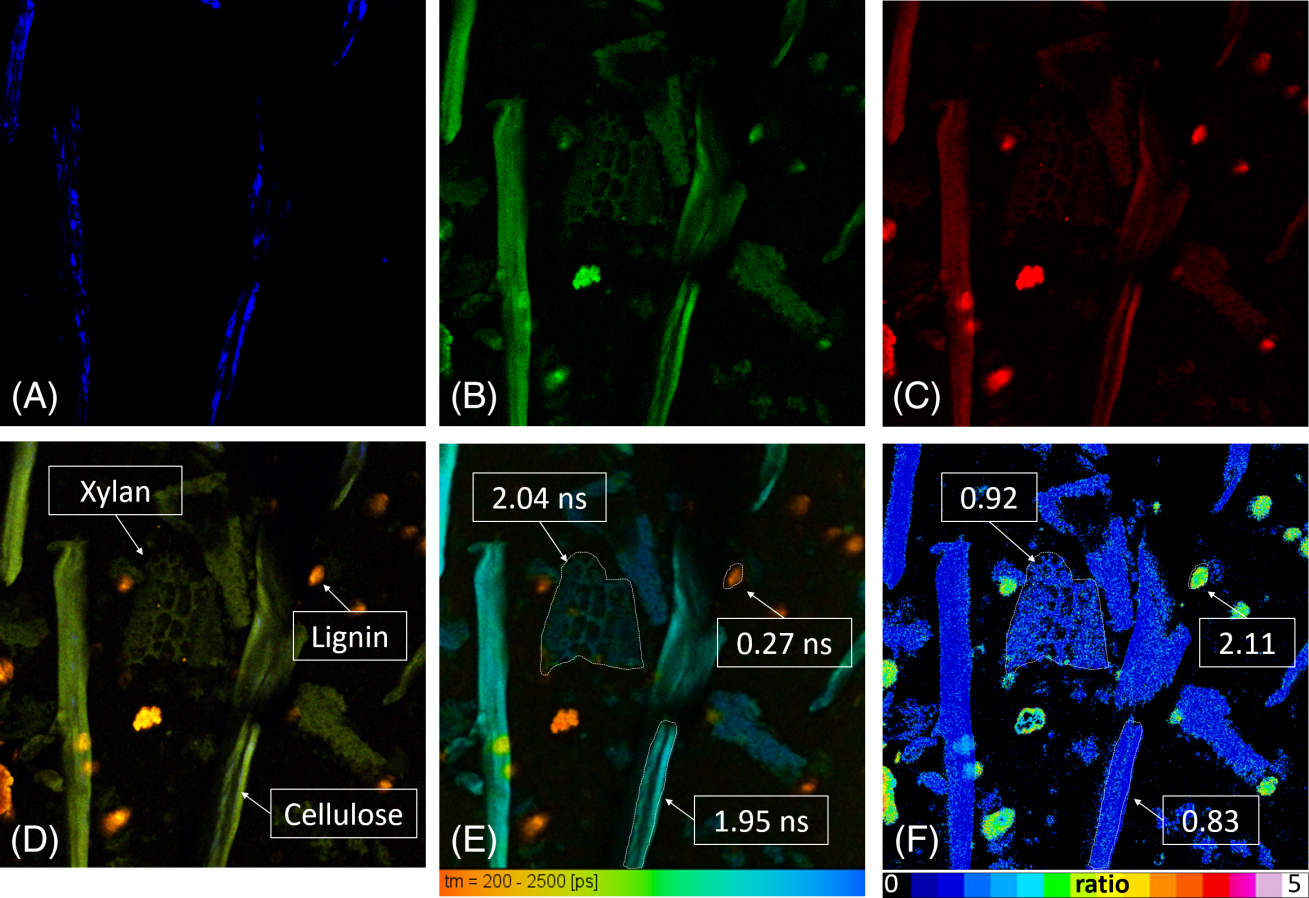
Composite biomass sample. A, SHG from cellulose, blue channel (400‐420 nm). B, Green channel (440‐525 nm). C, Red channel (550‐630 nm). D, Merged channels, cellulose and xylan appear green while lignin red. E, FLIM image, lignin (0.27 ns) has short lifetime and cellulose (1.95 ns) and xylan (2.04 ns) long. Average values are extracted from the corresponding ROIs. F, Fluorescence ratio (FR) image lignin has much higher ratio than cellulose and xylan

**Figure 3:**
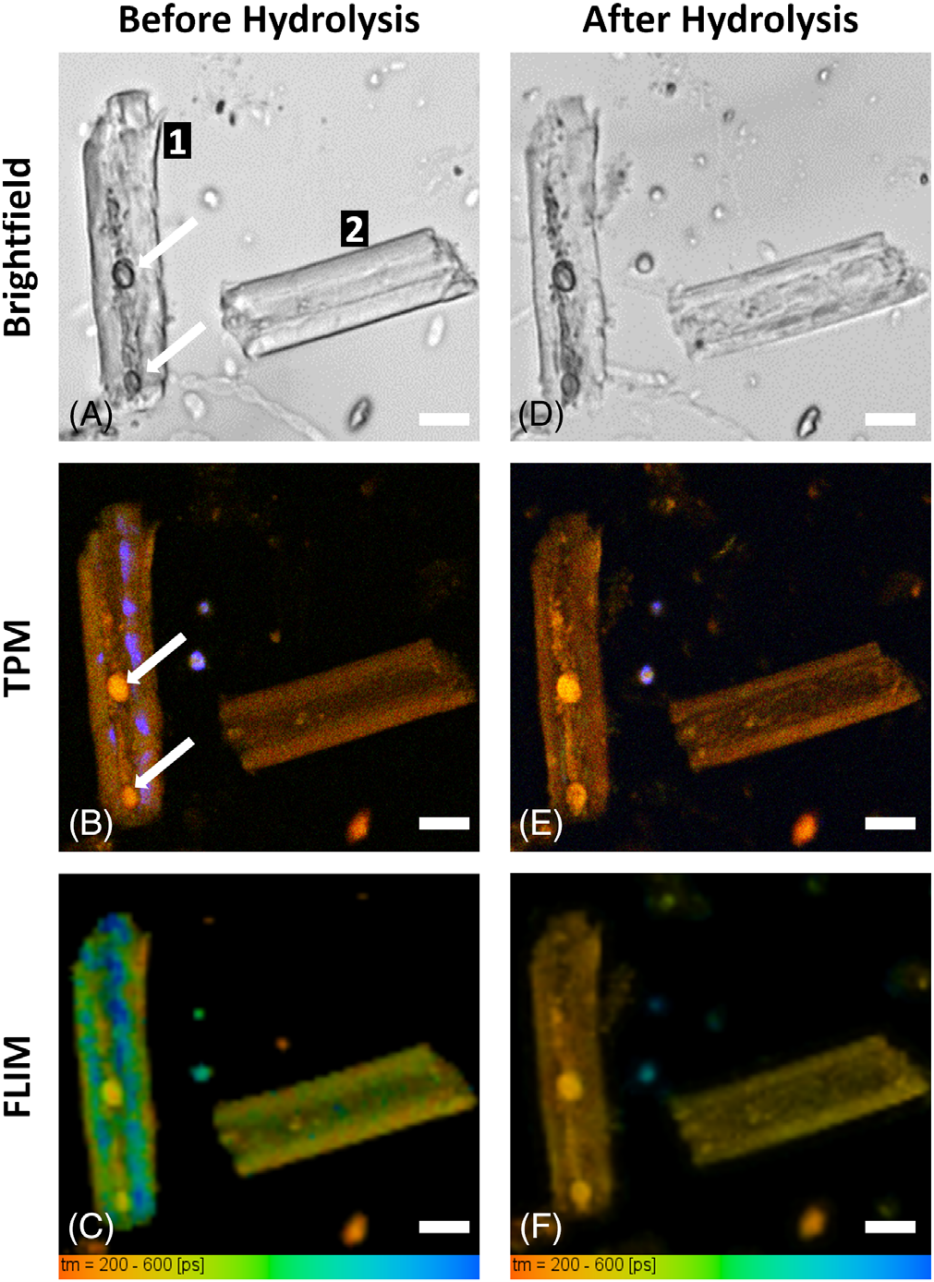
Monitoring pCS hydrolysis. A‐C, before hydrolysis and D‐F, after 18 hours of hydrolysis. A and D, brightfield; B and E, TPM; C and F, FLIM. Signs of hydrolysis are visible in the “after” images were particles became smaller and fragmented, fluorescence was red‐shifted and FLIM values decreased. (Scale bars: 10 μm)

**Figure 4:**
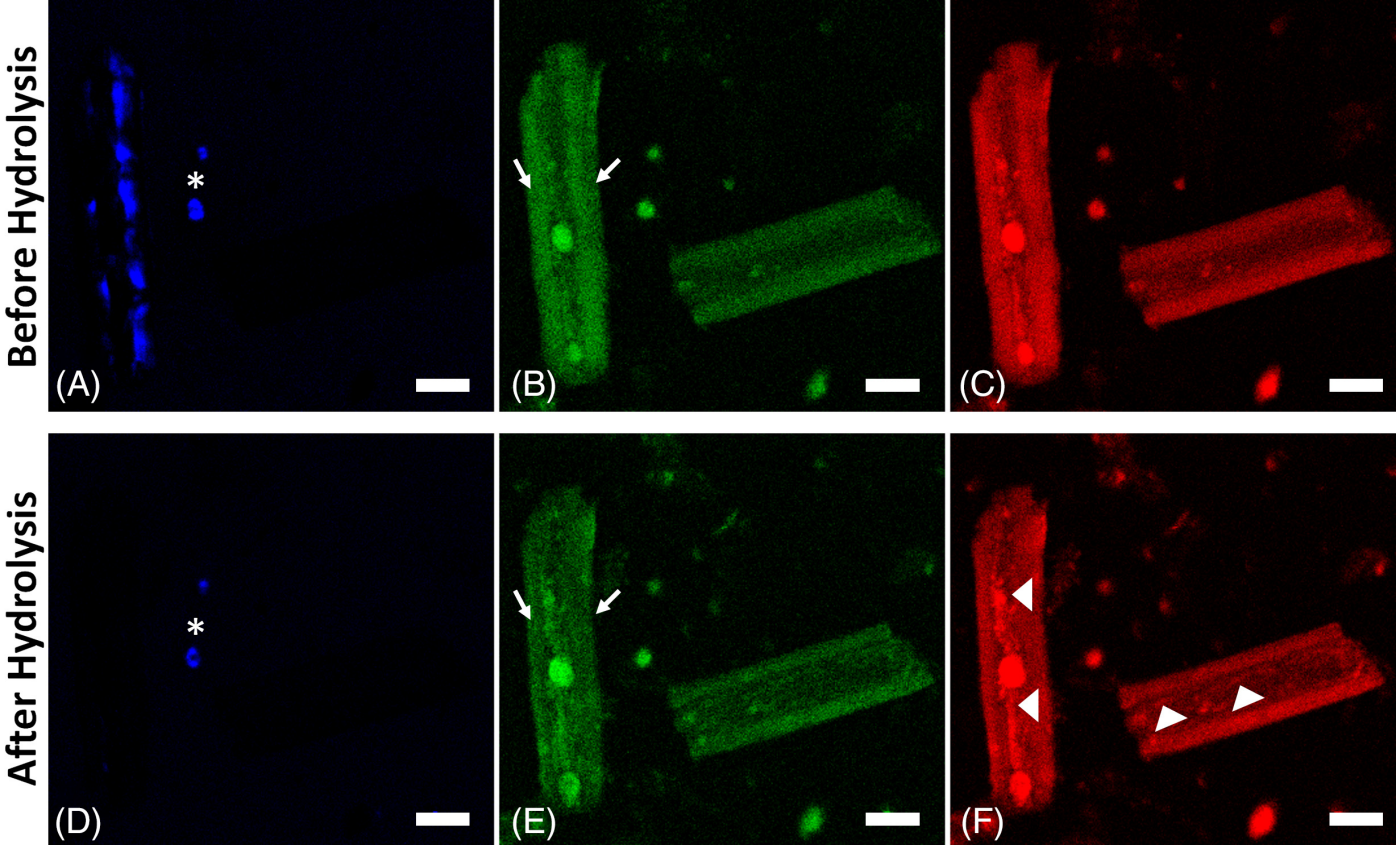
Spectral channels of TPM images. A‐C, Before and D‐F, after 18 hours of hydrolysis. A and D, Cellulose (SHG). Cellulose signal is absent in the after image. Impurities exhibiting SHG signal are indicted with star. B and E, Green channel: mainly cellulose‐hemicellulose: Arrows indicate regions of cellulose before hydrolysis which is absent after hydrolysis. C and F, Red channel: lignin. New lignin structures are indicated with arrowhead in F. (Scale bars: 20 μm)

**Figure 5:**
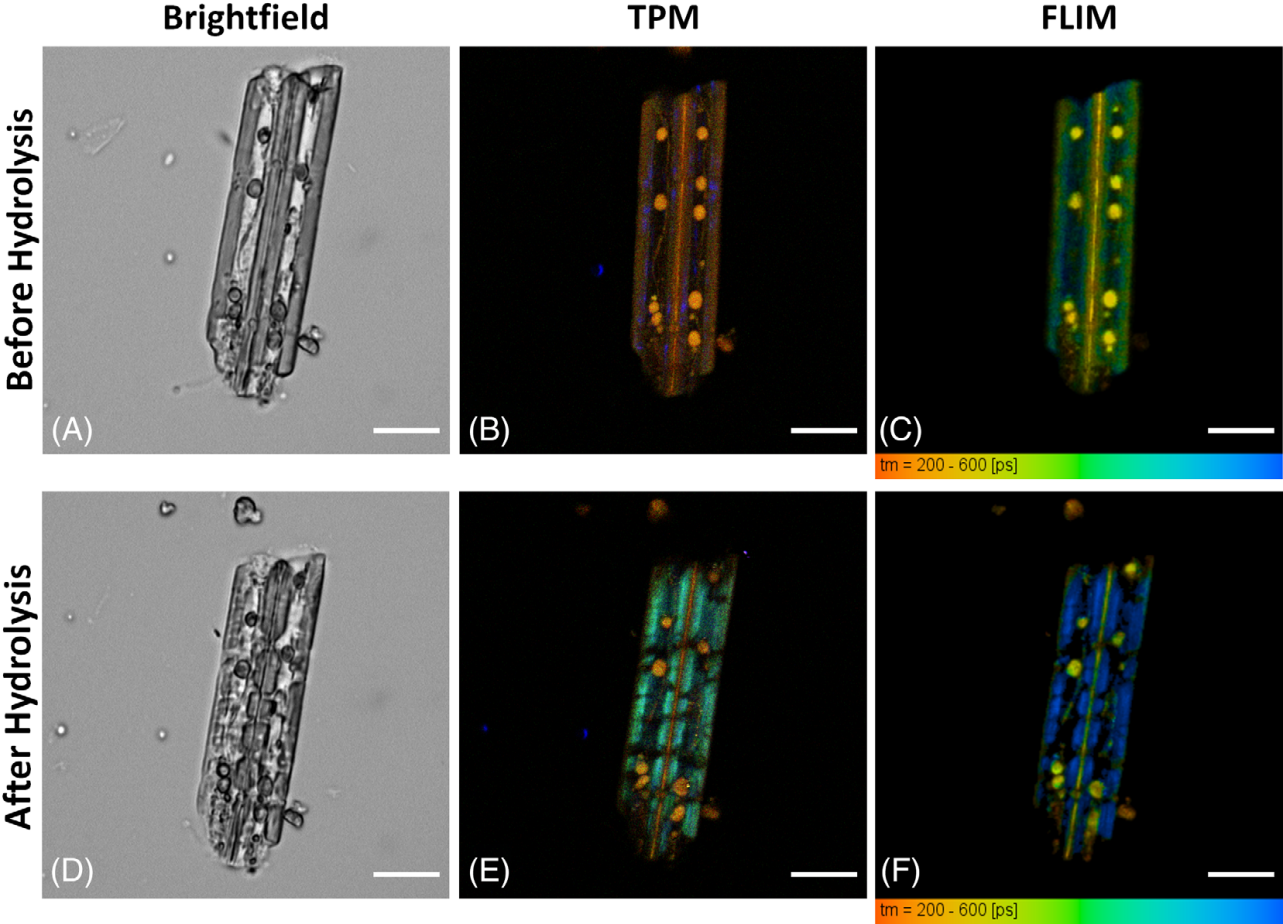
Monitoring pCS hydrolysis. A‐C, Before and D‐F, after 18 hours of hydrolysis. A and D, brightfield, B and E, two‐photon, C and F, FLIM. SHG from cellulose is visible in B while characteristic green patches have developed in E. In FLIM images lifetime increased after hydrolysis. This is attributed to enzyme binding on the particle. (Scale bars: 20 μm)

High‐resolution imaging with TPM can provide a wealth of information regarding morphology and chemical composition of individual particles. However, understanding and interpreting these results requires background knowledge on the properties on the individual cell wall components. The fluorescence properties of the individual building blocks of the plant cell wall were investigated and presented in Figures S1, S2, and S3. These results were first used to interpret the emission properties of a composite sample (resembling in composition a natural sample)—Figure 2 and afterward to interpret the results of natural biomass samples (pCS)—Figures 3, 4, 5. Finally, particle morphology and chemical composition before and after enzymatic hydrolysis were interpreted based on the properties of pCS particles and the properties of the major building blocks of the plant cell wall.

### 3.1 Composite biomass sample autofluorescence

In the Supporting Information, it was shown that the three major polymers of the plant cell wall all have strong autofluorescence signals. Biomass fluorescence depends on its local composition and is a combination of the emission of the individual components. In order to be able to interpret the biomass autofluorescence, we developed a general model for the emission characteristics of the individual cell wall building blocks based on the result in Figures S1M, S2J, and S3J. Cellulose and hemicelluloses have emission peaks at 510 and 520 nm regions, whereas lignin emission is more redshifted to the 580 nm region. Additionally, highly crystalline cellulose has strong SHG signal, while SHG is absent in hemicelluloses and lignins. Regarding lifetime, there is some variation in value for all three building blocks. Average lifetimes as calculated in section 1.4 *Average spectra and lifetimes* of Supporting Information was 2.16 ± 0.06 ns for cellulose, 2.01 ± 0.25 ns for hemicellulose, and 0.24 ± 0.01 ns for lignin. These observations are summarized in Figure 1A,B.

To produce high‐contrast fluorescence images and have good component separation, the intensity image acquisition was based on the results of Figure 1A. Crystalline cellulose has a distinct SHG signal; therefore, the forward detector was set to detect this narrow band signal in the 410 nm region. Cellulose and hemicellulose spectrally are similar; therefore, it was decided to detect both in the same channel in the 440 to 525 nm region. Spectrally is possible to differentiate lignin from the other components; therefore, a third detector was set to detect the lignin emission signal in the 550 to 630 nm bandwidth. With this configuration, we were able to detect the SHG signal of cellulose in the blue‐coded channel, the fluorescence emission of cellulose and hemicelluloses in the green‐coded channel, and lignin fluorescence in the red‐coded channel. To validate this detection configuration, a sample from a mixture of α‐cellulose, Birch xylan, and Protobind 1000 lignin was made with a ratio 4:3:3, respectively, to simulate the composition of a biomass sample. This sample was subsequently imaged, and the results are presented in Figure 2.

The three acquisition channels blue (Figure 2A), green (Figure 2B), and red (Figure 2C) are presented separately. The merged image is presented in Figure 2D. SHG originating specifically from crystalline cellulose is visible in Figure 2A. In the green channel, the fluorescence emission of cellulose and hemicellulose is more prominent. Crystalline cellulose can be identified by its SHG signal, while the presence of hemicellulose is identified mainly by its morphological appearance in the green channel and the absence of SHG. In the red channel, lignin appears as bright red aggregates. Fluorescence spectra of all components are broad and as a result spectral cross‐talk is present. In the merged image (Figure 2D), however, different components are much easier to differentiate based on their color, as cellulose and hemicellulose appear green and lignin red. Moreover, crystalline cellulose is identified by the characteristic SHG signal (blue). In the case where cellulose and lignin colocalize, the result is yellow as seen in Figure 2D. Finally, in the FLIM image (Figure 2E), cellulose (1.95 ns) and hemicellulose (2.04 ns) can be discerned by their long lifetime (blue coded), while lignin (0.25 ns) has very short lifetime (red coded). The lifetime values are the averages of the indicated regions of interest (ROIs) in Figure 2E. The different components are identified by their morphology, spectral emission, lifetime, and presence or not of SHG. Even though this composite sample is not a natural sample, it demonstrates well that morphology and fluorescence properties of different cell wall components can be visualized and identified with good resolution, and each of them has a distinct and considerable contribution to the total autofluorescence signal from biomass.

The capability of extracting compositional information about the sample based on the average lifetime was explored. The average lifetime for Figure 2E is 1.2 ± 0.2 ns, and the average lifetime of composite samples is 0.9 ± 0.2 ns. If we assumed that such a composite sample was composed of cellulose, hemicellulose, and lignin in 4:3:3 ratio and each of them had equal quantum yields and extinction coefficients, then the theoretical lifetime of such a sample would be the mean lifetime of these three components weighted by their concentration. Assuming 40% cellulose concentration with 2.16 ns lifetime, 30% hemicellulose with 2.01 ns, and 30% lignin with 0.24 ns then the average theoretical lifetime value would be 1.54 ns. This is far from the 0.9 ns average lifetime of composite sample measured here. The average intensity level of lignin as defined in the ROI of Figure 2D is 147, that of cellulose 53, and hemicellulose 24. In general, lignin is much brighter than the other two building blocks of biomass and images such as Figure 2E are not safe to extract exact compositional information about the sample, as less bright components might not be visible in the image. However, changes in chemical composition will be reflected in the lifetime, and this could be very well used to identify compositional changes after pretreatment and during enzymatic hydrolysis.

Fluorescence ratio (FR) Figure 2F is simply the division, pixel by pixel, of the red channel, which contains mainly the lignin emission, over the green channel, which contains mainly the emission of the cellulose‐xylans. This creates a new image where lignin‐rich and cellulose‐hemicellulose‐rich areas are highlighted with different colors, the higher the ratio the higher the relative lignin content. Characterization of a whole sample based on FR would not be practical due to the nonlinear dependence of fluorescence to the excitation intensity and the inhomogeneity of corn stover samples. This ratio can become particularly useful when analyzing emission changes of an individual particle.

### 3.2 *In situ* imaging of hydrolysis of pretreated corn stover particles

The changes of autofluorescence properties of biomass samples during enzymatic hydrolysis were used to monitor morphological and chemical composition changes that take place during hydrolysis. Investigation was based on individual pCS particles. To achieve this, a custom chamber with a mixture of pCS and enzyme cocktail (DSM, Delft) was placed under the microscope, and the effect of hydrolysis was imaged *in situ* on the same particles for 18 hours. Characteristic cases are presented in Figures 3, 4, 5. Brightfield, TPM, and FLIM images were acquired.

In the brightfield images, the general morphology of the biomass particles can be seen. Particles with a cane‐like structure were commonly observed. In the TPM images, the three detection spectral channels are overlaid in a single image (Figure 3B,D). Particles have an intense yellow‐brown color, suggesting that the contribution of lignin is higher than the emission signal from cellulose and hemicellulose. In these images, the presence of SHG offers a direct evidence of crystalline cellulose presence. However, it should be noted that SHG absence is not necessarily evidence of cellulose absence. The autofluorescence decay properties are presented in the FLIM images (Figure 3C,F) The fluorescence lifetime of these particles is characterized by very low values, but this can be well correlated with the findings of spectral images where the contribution of lignin fluorescence is already significant.

Comparing the images before and after hydrolysis, it is possible to observe changes in particle size and morphology. Particle 1, before hydrolysis (Figure 3A), had a cane‐like shape, with two distinct round structures (arrows) in the inner part. After hydrolysis (Figure 3D), it was more transparent, it appeared to have less texture and was smaller in size. Both its ends appeared to be disintegrating, and new features appeared along its central axis. The round structures appeared unaffected and at the same position. Particle 2, before hydrolysis had well‐defined thick side walls and after they were thinner and less homogeneous and appeared more fragmented. Also, some new features appeared in the center of the particle which were the result of the collapse of its sidewall. Both particles' cross‐sectional surface had decreased after hydrolysis, that is, 10% for particle 1 and 8% for particle 2 (measured as described by Kapsokalyvas *et al*.^38^), which is an additional indication for cellulose removal.

Comparing the images in Figure 3 with Figure 2, we observe that the different components do not appear separately, but all present in the individual particles making interpretation of the image more difficult. In the TPM images spectroscopic changes can be observed. Before hydrolysis, particle 1 had a strong SHG signal, indicating cellulose presence and the two round structures (arrows), which were also observed in brightfield, had intense yellow color. In the separated spectral channels (Figure 4), these round structures were brightest (almost to the level of saturation) in the red channel (Figure 4C). Spectrally they correspond to lignin (Figure 2D); therefore, we concluded that these were lignin droplets. These lignin droplets appeared unaffected and at the same position after hydrolysis (Figure 3E). Moreover, SHG signal disappeared; therefore, cellulose had been hydrolyzed. Lignin molecules appeared to be aggregating in the central axis of the particle forming a kind of lignin backbone. Additionally, the side wall appeared more redshifted after hydrolysis because of cellulose removal. Particle 2 was also hydrolyzed. Before hydrolysis, thick well‐formed sidewalls were observed. These sidewalls were still present after hydrolysis, although they were smaller and slightly redshifted. Overall, particle 2 appeared more fragmented, and new features corresponding to lignin were observed in the inner part. SHG was not observed in this particle.

Changes were also observed in the FLIM images (Figure 3C,F). The average lifetime of particle 1 before hydrolysis was 390 ± 36 ps and after it decreased to 258 ± 18 ps. This is also visible by the change of the color coding from green to yellow in Figure 3C,F. This corresponds well with the observation that cellulose and hemicellulose, which have longer lifetimes (Figure 2F), had been removed and no longer contributed to the autofluorescence signal, while lignin which has very low lifetime, accumulated and accounted more for the autofluorescence signal of this particle. Lignin droplets, which were also observed in Figure 3B,E, exhibited very low lifetime values, characteristic of lignin (see Figure 2F). Similar observations can be made on particle 2 were average lifetime decreased after hydrolysis from 312 ± 14 ps to 286 ± 8 ps and is visualized by the color change in Figure 3C,F. The change in lifetime was not as high compared to particle 1, and this could be correlated with the observation that particle 1 contained more cellulose (SHG signal in Figure 3B) which was removed during hydrolysis and therefore experienced bigger compositional change.

TPM images have higher resolution compared to brightfield and FLIM. Brightfield has good depth of focus but lacks sectioning capability. FLIM in principle has the exact same capabilities as TPM, but due to the nature of the acquisition and the limited pixel number that can be used, the full resolution was not exploited. Therefore, detailed morphological observations were made on the TPM images. Since there is spectral overlap between the cell wall building blocks, as was observed on the composite biomass sample (Figure 2), some features are better visible in the overlay images (Figure 3B,D). However, more detailed observations can be made on the spectral images. The individual spectral channels from images in Figure 3B,D are presented in Figure 4. In Figure 4A,D, crystalline cellulose based on its SHG signal can be observed. After hydrolysis, cellulose has been removed as no SHG from particle 1 was observed. However, there were two particles (marked by “star” in Figure 4A,B) that exhibited SHG signal before and after hydrolysis. These particles are not biomass particles but impurities, such as minerals and soil particles, that were collected along with corn stover from the field. Such impurities can exhibit SHG signal, but they are not affected by hydrolysis. The SHG signal of the impurities in Figure 4 remained unaffected after hydrolysis; however, SHG from cellulose disappeared, which offers an additional indication that indeed cellulose was hydrolyzed. In Figure 4, the spectral contribution from cellulose‐hemicellulose (green channel) and lignin (red channel) can be studied. In the overlay of the images, lignin gives a yellow‐brown color as observed in the composite sample (Figures 2D and 3B,D). Lignin droplets were well visible in the inner part of particle 1 before and after hydrolysis. New lignin structures, (marked by “arrowheads” in Figure 4F) were visible after hydrolysis in both particles 1 and 2, respectively. As cellulose was removed and the side wall collapsed, lignin molecules aggregated in the inner part of the particles. Lignin accumulation and aggregation have been observed in the past.^39^ Cellulose and hemicellulose can be studied in the green channel in Figure 4B,D. The strong signal before hydrolysis (arrows in Figure 3B) on the side walls of particle 1 was absent after hydrolysis, which is another indication of cellulose removal. We expect that decrease of green signal (due to cellulose removal) will cause the increase of FR. In particle 1 (see Table 1), there was significant FR change (19%), while for particle 2, none was observed. We speculate that due to the absence of SHG in particle 2, cellulose content was lower; therefore, compositional changes were not as big as in particle 1. In Table 1, we observe that particle 1 exhibits big changes in all measured parameters (surface reduction: 10%, FR: 19%, and FLIM change: 34%), whereas for particle 2, corresponding values are all moderate (8%, 0%, 8%, correspondingly). Therefore, we conclude that for reasons that we are not able to determine in this study, particle 1 has been more hydrolyzed than particle 2. This is also consistent with the fact that different particles are hydrolyzed at different rates and different extent. Moreover, FLIM seems to be the most sensitive parameter.

**Table 1:** Summary of changes observed during hydrolysis of particles 1 and 2

		Before	After	Change (%)
Particle 1	Surface (μm^2^)	745.2 ± 0.6	668.3 ± 0.6	−10
	RATIO	2.1 ± 0.6	2.5 ± 0.7	19
	FLIM (ps)	390 ± 14	258 ± 8	−34
Particle 2	Surface (μm^2^)	640.1 ± 0.6	590.1 ± 0.6	−8
	RATIO	2.64 ± 0.8	2.64 ± 0.8	0
	FLIM (ps)	312 ± 36	286 ± 18	−8

Based on the images of Figures 3 and 4, it is concluded that the effect of hydrolysis was manifested with particle size reduction, side wall collapse, loss of cellulose (SHG) signal, red shifting of the autofluorescence emission (increase of FR), and autofluorescence lifetime decrease. Quantification of these observations is summarized in Table 1. These observations can be used to assess the effect of hydrolysis on a biomass particle. These results cannot be correlated to absolute values of chemical composition of the sample but can serve as an index to monitor the relative compositional change. These are preliminary results that demonstrate the possibilities of the developed methodologies. Further examination of a larger sample size and statistical analysis could provide information on the changes that take place on a bigger scale and reveal the behavior of the whole sample.

TPM has been used for imaging lignocellulosic samples to demonstrate morphological changes after pretreatment or during hydrolysis. In sugar cane bagasse samples, it was shown that after chemical bleaching, which removes lignin, fibril packages of bagasse sample partially lost their longitudinal orientation.^28^ *In situ* enzymatic hydrolysis imaging of pretreated softwood revealed that SHG signal decreased at increasing hydrolysis, indicating the conversion of cellulose to monosugars^29^ as it was also shown here. Additionally, the autofluorescence signal was shown to decrease after hydrolysis; however, the origin of the signal was not attributed to a specific source. In this study, we demonstrated that autofluorescence is a mixture of all the basic polymers of the plant cell wall and associated morphological changes with chemical changes—cellulose removal and lignin aggregation (Figure 4).

### 3.3 Binding of enzymes to substrate

It has been suggested that enzyme binding to noncellulose molecules, such as lignin, can take place during enzymatic hydrolysis and that this leads to decreased hydrolysis efficiency.^39^, ^40^ In the present study, enzyme activity on corn stover was visualized. A characteristic case is presented in Figure 5. Examination of the brightfield images reveals that morphology of the particle, before and after hydrolysis (Figure 5A,D), changed and it appeared more degraded and fragmented, similar to particles 1 and 2 (Figure 3A,D), and particle size surface decreased 4%. Morphology changes during hydrolysis can be also observed in the time lapse movie in Supporting Information Video S1. Therefore, it has been hydrolyzed. Morphological changes are clearly observed in this video. We pursued to acquire such time lapse videos also in fluorescence mode. Because the particles were in a heated environment (50 °C), and they were not attached to the glass but merely suspended, there were cases that after TPM imaging the particles would relocate outside the field of view, therefore compromising the whole experiment. For this reason, for the present study, we chose to observe only the before and after states in fluorescence mode. Before hydrolysis in the TPM image (Figure 5B) also SHG from cellulose was detected. However, after hydrolysis (Figure 5E), fluorescence in the green channel increased and characteristic green patches became visible. It would be expected that signal in the green channel would decrease with hydrolysis (as in Figure 3) due to the removal of mainly cellulose. FR in this case decreased 54%, but this is due to the increase of intensity in the green channel, not decrease in the red channels and although parts of the particle seem to be removed, just lignin, removal is not enough to explain this result. These green patches were attributed to enzyme binding to lignin. The enzyme cocktail is autofluorescent, but its signal is negligible compared to biomass autofluorescence. However, in cases where there is strong binding, it is possible that locally the concentration of enzymes could increase greatly, producing this bright signal. Spectrally, the peak in the green part of the spectrum coincides with the peak of enzyme autofluorescence. Lifetime (Figure 5F) also increased after hydrolysis. Since lifetime of the enzyme cocktail is long (1722 ± 122 ps, see Supporting Information section 1.5), its presence inside the particles compartments leads to the overall increase lifetime of the particle from 370 ± 94 ps before to 583 ± 157 ps after. This phenomenon was observed only in some of the particles that were analyzed, and the reason is not yet clear. In general, several such particles were observed in the hydrolysis chamber at the end of hydrolysis. However, since there was no prior knowledge that this phenomenon would occur, we only had the opportunity to observe the before and after results in only a handful of them. Moreover, it does not correlate with specific structures. Bound enzymes do not contribute to the hydrolytic process anymore, thus limiting the conversion efficiency, especially at low enzyme loading as in industrial applications. Further analysis of such particles would be valuable for improving our understanding of recalcitrance in corn stover. It has been suggested that certain agents such as bovine serum albumin (BSA)^41^ can act as anti‐inhibitory factors preventing the binding of enzymes to lignin. With the microscopic methodology developed here, the anti‐inhibitory effect could be observed directly on individual particles, and fast screening of different anti‐inhibitory agents could be performed.

## 4 CONCLUSIONS

The autofluorescence properties of biomass particles and plant cell wall components were investigated with TPM. Major building blocks of plant cell wall, cellulose, hemicellulose, and lignin exhibited strong autofluorescence signal with characteristic properties. These properties can be used for spectral and lifetime‐based separation of lignin from cellulose and hemicellulose. Monitoring of hydrolysis *in situ* was made possible, and the change in the autofluorescence response of corn stover particles was visualized. Relative changes in chemical composition gave rise to changes in autofluorescence response, and this property could be used to investigate the effect of hydrolysis on individual particles. It was used to assess the different degrees of hydrolysis between different particles and also used to identify enzyme binding to the substrate. Such observations can be used to investigate the potency of different enzyme cocktails on specific particles that are considered recalcitrant therefore offering an alternative and more direct screening method. Additionally, *in situ* hydrolysis imaging could be used to visualize the effect of anti‐inhibitory agents directly on individual model or biomass particles and thus offering an accurate and direct method for investigating the properties of such agents.

## CONFLICT OF INTEREST

Ilco Boogers, Maaike Appeldoorn, and Joachim Loos are employees of Royal DSM N.V.

## Supporting information


**Data S1** Morphochemical characterization of individual plant cell wall components.

**Video S1** Time lapse video of in‐situ enzymatic hydrolysis of a pCS particle.
