# Supplementary material for: Quantification of morphochemical changes during in situ enzymatic hydrolysis of individual biomass particles based on autofluorescence imaging
Source: Biopolymers. 2019 Dec 23;111(3):e23347. doi: 10.1002/bip.23347 (PMC7154748; doi:10.1002/bip.23347)
Supplement: Supplementary file 1 — Data S1 Morphochemical characterization of individual plant cell wall components. [file BIP-111-e23347-s002.pdf]

Supporting Information

Quantification of morphochemical changes during in-situ enzymatic hydrolysis of individual biomass particles based on autofluorescence imaging

Dimitrios Kapsokalyvas [*a], *Joachim Loos*[b], *Ilco A.L.A. Boogers*[c], *Maaike M. Appeldoorn*[c] *Mirjam A. Kabel*[d], *and Marc Van Zandvoort* [e, f]

[a] Department of Molecular Cell Biology, GROW, CARIM, Maastricht University, Maastricht, 6229 ER, The Netherlands

[b] Royal DSM N.V., Materials Science Center, 6167 Geleen, The Netherlands

[c] Royal DSM N.V. Biotechnology Center, Delft, 2613 AX, The Netherlands

[d] Laboratory of Food Chemistry, Wageningen University, Wageningen, 6708 WG, The Netherlands

[e] Department of Molecular Cell Biology, CARIM, GROW, MHeNs, NUTRIM, Maastricht University, Maastricht, 6229 ER, The Netherlands

[f] Institute for Molecular Cardiovascular Research (IMCAR), RWTH Aachen University, Aachen, 52704, Germany

[*] d.kapsokalyvas@maastrichtuniversity.nl

1 Cell wall components

Goal of this study was to use the autofluorescence signal of plant cell wall components to identify their localization in biomass particles i.e. pCS and to investigate the chemical changes during hydrolysis. Since corn stover is mainly composed of cellulose, hemicellulose (mainly xylan), and lignin, their autofluorescence spectra and lifetime were investigated individually in this section. The validity of the model developed for interpreting the fluorescence signal from biomass was tested in a composite biomass sample.

1.1 Cellulose

Naturally, cellulose occurs in both crystalline and amorphous form in the plant cell wall. Amorphous cellulose can adsorb water and is easier hydrolyzed compared to the crystalline form [1]. Moreover cellulose crystallinity decreases with pretreatment [2]. Reduction of cellulose crystallinity with pretreatment has been associated with higher yields, however there seems not to be always true as there have been cases were higher crystallinity resulted to higher digestibility [3]. The properties of celluloses of varying crystallinity were examined. Celluloses with high crystallinity index such as Avicel, and α-Cellulose, as well as amorphous celluloses such as D4MRes, and regenerated amorphous cellulose (RAC) were investigated. Using NMR, [4] measured a crystallinity index of 56.7 for Avicel and 41.5 for α-Cellulose. Both the D4MRes and RAC are considered amorphous due to the conditions used in the preparation.

In Figure S1, representative images of the various cellulose samples are given. In the brightfield images (Figures S1A-S1D), the general view and the texture of the sample can be seen. Cellulose samples are generally transparent. Avicel and α-Cellulose form cylinder like structures and have a ‘crystalline’ texture, while the amorphous samples D4Mres and RAC are also transparent but don’t have a distinctive structure. The TPM autofluorescence signal is

presented in Figures S1E-S1H. All samples appear green, with emission maximum at 510 nm. Some particles have a local characteristic SHG signal coded with blue color. Cellulose is a non-centrosymmetric molecule, and in its crystalline form can produce SHG signal. SHG is a coherent phenomenon that becomes significant and observable when the level of organization of the material is high [5] such as in a crystal. Loss of order on the structure of the material leads to decrease and eventually to absence of SHG signal[6], e.g., in amorphous structures. SHG signal from cellulose has been shown to be polarization dependent as with all non-centrosymmetric molecules, and its signals was shown to decrease exponentially during hydrolysis [7]. Cellulose SHG properties were also shown to be dependent on crystallinity as increased swelling with ionic liquid decreased SHG signal [8]. Indeed, we observed that the SHG signal from cellulose increased with increasing crystallinity. Avicel and α-Cellulose had extended regions with SHG. D4Mres and RAC, even though considered to be fully amorphous samples, still exhibited some regions with SHG signal. It is possible that some cellulose molecules re-crystalize when soaked in water [9] which could explain the crystalline pockets observed. In the FLIM images (Figures S1I-S1L) the fluorescence lifetime decay is visualized. There is a variation in lifetimes between samples and within particles of the same sample.

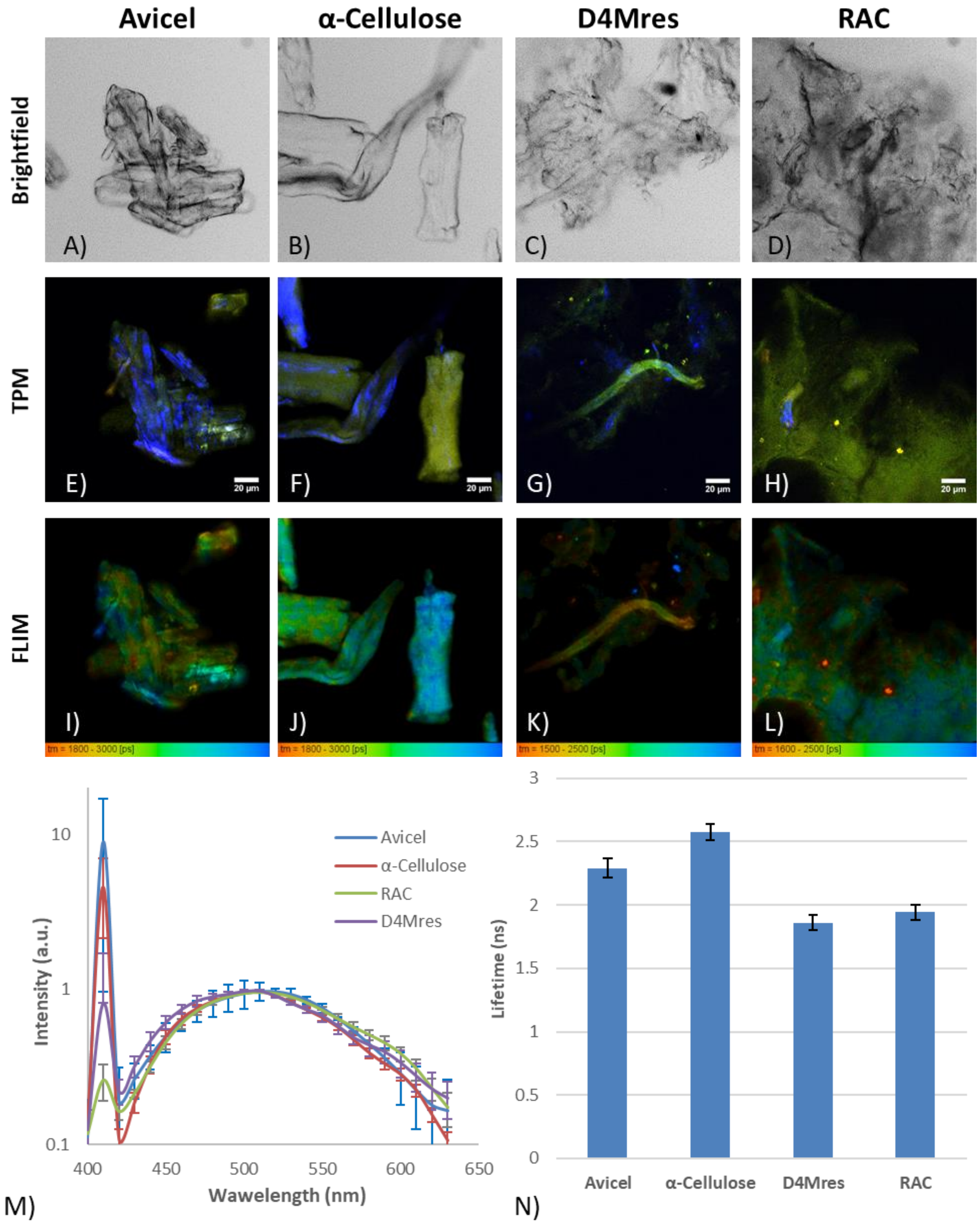

Figure S1. Images of different celluloses. **(A-D)** bright-field, **(E-H)** TPM, **(i-l)** FLIM images. Avicel images (A,E,J), α-Cellulose (B,F,J) D4Mres amorphous cellulose (C,G,K), and RAC amorphous cellulose (D,H,L). **(M)** Autofluorescence spectra of different celluloses all exhibit a peak at 510 nm. Avicel and α-Cellulose have strong SHG signals. **(N)** Lifetime values. Highly crystalline celluloses have slightly higher lifetimes compared to amorphous. Error bars correspond to the standard deviation.

The average spectra of the different samples and corresponding average lifetimes are presented in Figures S1M and S1N, respectively. Avicel has much stronger SHG signal compared to α-Cellulose. Both have significantly higher SHG signal compared to the amorphous cellulose samples. On the other hand, autofluorescence was very low in the very crystalline Avicel, whereas it was much higher in the α-Cellulose. Interestingly autofluorescence levels were also low for RAC, which is derived from treated Avicel, while D4MRes autofluorescence levels were similar to α-Cellulose. Intensity levels were only studied qualitatively. In all cellulose samples examined, we observed an emission maximum around 510 nm. Regarding lifetime (Figures S1I-S1L), there was some variability. The more crystalline samples had higher lifetimes, Avicel 2.29 ± 0.07 ns and α-Cellulose 2.57 ± 0.06 ns, while the amorphous samples had shorter lifetimes (D4Mres 1.86 ± 0.06 ns and RAC 1.94 ± 0.06 ns). Cellulose fluorescence lifetime was decreased in amorphous samples, however the peak of autofluorescence did not shift. In the amorphous samples, cellulose molecules are most likely more in contact with the solution medium, which might influence their fluorescence decay rate, but does not cause a shift in the autofluorescence spectrum. However, it should be noted that spectral resolution (10nm) of the measurement is not enough to detect very small shifts.

The origin of cellulose fluorescence is not completely understood. Isolated cellulose does not fluorescence but when it forms bigger complexes like in its crystalline from it can become fluorescent[10]. Cellulose has a broad emission spectrum with an emission peak at 410- 430 nm when excited with UV light (310-365) nm [10–12]. However its emission peak will shift at longer excitation wavelengths, as expected and as has been shown[12]. We measured the crystalline

cellulose fluorescence lifetime (avicel and α-Cellulose) 2.43 ns which is very close to the reported value of 2.39 ns [10]. Therefore our reported values are in accordance with literature.

1.2 Hemicelluloses

Hemicelluloses interconnect cellulose fibers. There are various types of hemicelluloses, most common being the xylans. Fluorescence properties of wheat arabinoxylan (WAX), birch xylan, and oat spelt xylan (OSX) were analyzed. Representative images are visualized in Figure S2. WAX had very good solubility in water and therefore produced homogeneous images (Figure 2A), appearing green in the TPM image (Figure S2D), with long and homogeneous lifetime in the 2.3 ns range (Figure S2G). Birch xylan could also be dissolved adequately in water, but occasionally non-dissolved particles could be found (Figure S2B). These had a string like texture. In the corresponding TPM, (Figure S2E) these particles appear green. In Figure S2H the corresponding FLIM image is given. OSX, (Figures S2C, S2F, and S2I), which is composed mainly of linear crystalline xylan [13], had a more compact texture, visible as green clusters in the TPM image, with slightly shorter lifetime (Figure S2I) compared to WAX and birch xylan. In Figures S2J and S2K the average spectra and lifetimes are presented. WAX had an emission peak at 510 nm and an average lifetime of 2.26 ± 0.03 ns. The emission peak of birch xylan was at 520 nm (Figure S2J) with slightly broader FWHM compared to WAX and an average lifetime 1.76 ± 0.037 ns (Figure S2K). OSX had an emission peak at 510 nm and shorter fluorescence lifetime (1.22 ± 0.05 ns). WAX and OSX had higher content of arabinoxylans, than Birch (85% xylose) which might explain the small difference in spectral emission (WAX and OSX: 510 nm, Birch xylan: 520 nm). However, lifetime between WAX and OSX were distinctively different and this might be affected by the local environment of each sample. OSX was less pure (80% xylose and arabinose, 15% glucose) compared to WAX (95% arabinose

and xylose), and potentially could have affected the fluorescence transition rates of OSX. Conclusively, for xylans, emission peaks did not vary significantly (510-520 nm), while there was significant variability in lifetime with values between 1.2 -2.3 ns. Xylans have similar emission properties compared to cellulose, however no SHG signal was observed.

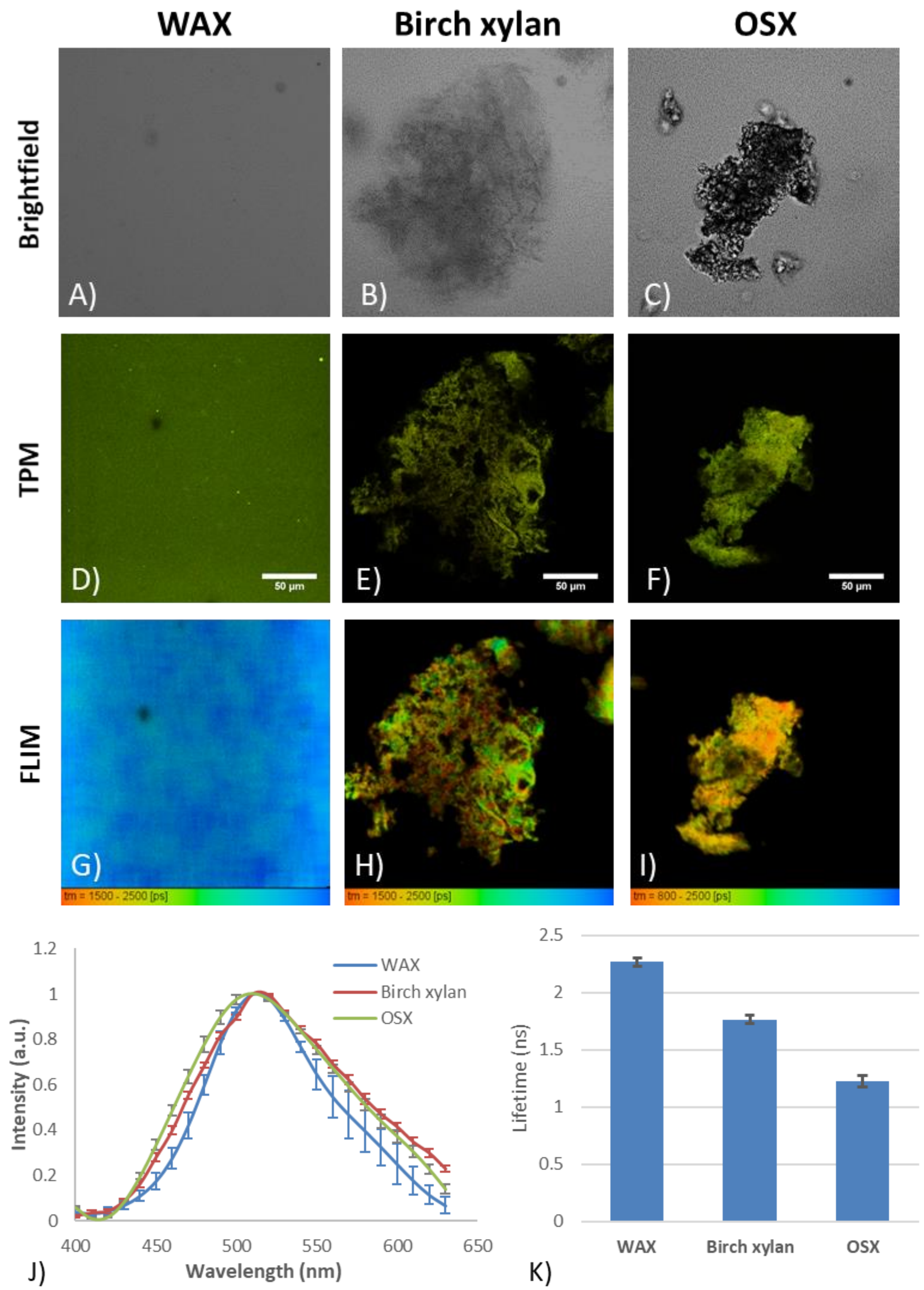

Figure S2. Images of xylans. (A-C) bright-field, **(D-F)** TPM **(G-I)** FLIM images. Birch xylan images (A, D, G), WAX (B, E, H), and Oat spelt xylan (OSX) (C, F, I). **(J)** Autofluorescence spectra of different xylans, emission peak between 510 (WAX and OSX) and 520 nm (Birch xylan). **(K)** Corresponding lifetime values range from 2.3 ns (WAX) to 1.8 ns (Birch xylan) and 1.2 ns (OSX).. Error bars correspond to the standard deviation.

1.3 Lignin

Lignin is mainly found on the secondary wall of the plant cell wall. It provides rigidity on the cell wall as also impermeability to water, therefore supporting the water transport in plants. It is hydrophobic and therefore not soluble in water. Lignin samples of different origins were analyzed: Alcell, Protobind 100, and Indulin AT. Samples were immersed in water and formed aggregates or small particles that were well visible under the microscope. Representative images are presented in Figure S3. In the brightfield images (Figures S3A-S3C), it is visible that all samples form aggregates of variable sizes. Lignin did not dissolve in water as expected. In the TPM images all lignin samples appeared red (Figures S3D-S3F) and in the FLIM images (Figures S3G-S3I) exhibited short lifetimes. Average spectra and lifetimes are presented in Figures S3J and S3K. Lignin emission was red-shifted compared to cellulose and hemicellulose. Alcell had an emission peak at 600 nm, Indulin AT at 560 nm and Protobind 1000 at 590 nm. All lignin samples measured had very short lifetimes between 230-260 ps. Our findings on lignin lifetime correlate well with literature [14,15]. Conclusively, lignin spectrally is red-shifted compared to cellulose and xylans and has a significantly shorter lifetime.

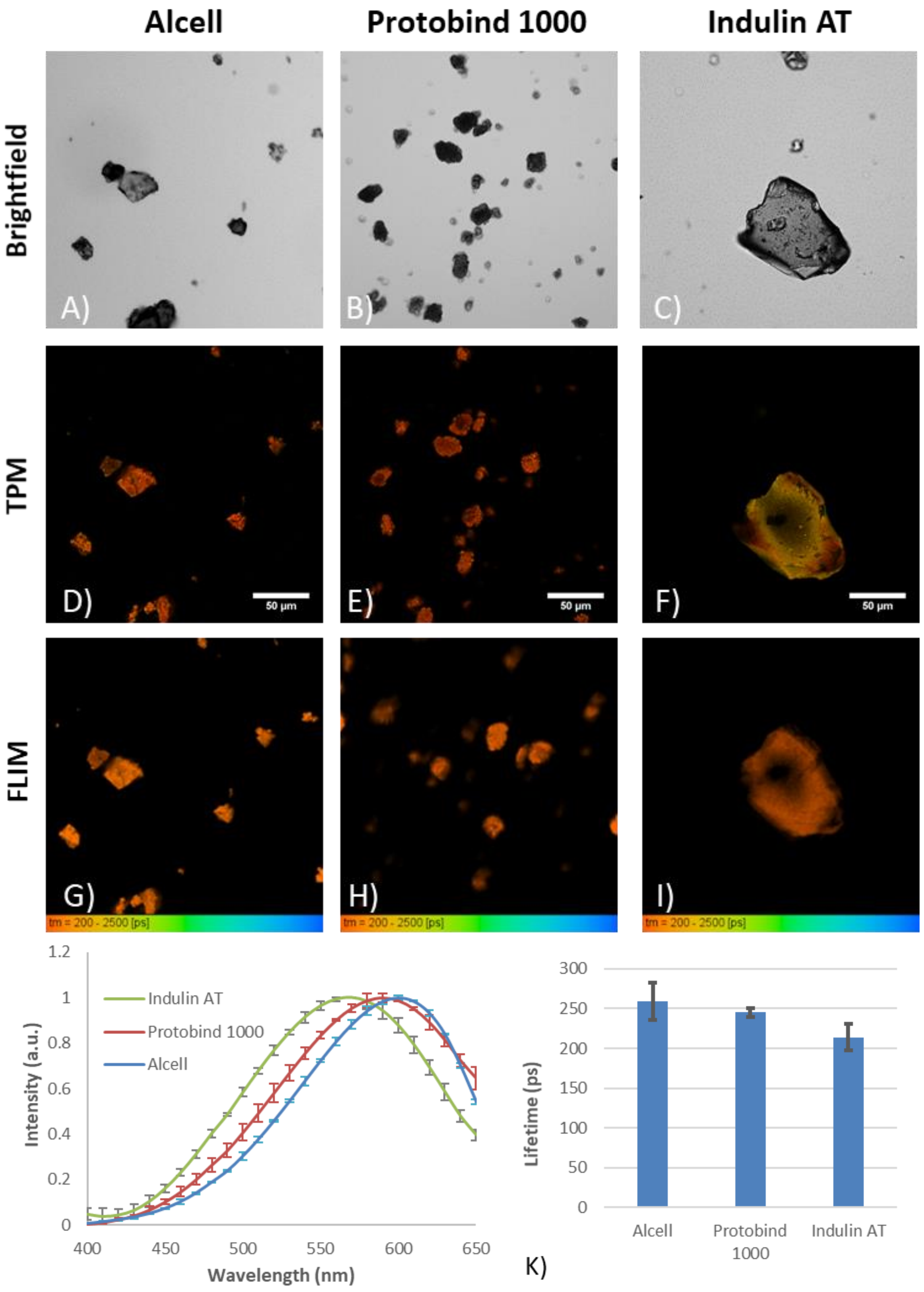

Figure S3. Images of different lignins. **(A-C)** bright-field, **(D-F)** TPM **(G-I)** FLIM images. Alcell images **(A, D, G)**, Protobind 1000 **(B, E, H)**, Indulin AT **(C, F, I)**. **(J)** Autofluorescence spectra of different lignins, emission peaks between 560-600 nm, **(K)** Lifetime values. Lignin average lifetime is very short between 0.21-0.26 ps.

1.4 Average spectra and lifetimes

The average spectra of all celluloses, hemicelluloses, and lignins are presented on the graph below. Cellulose is the average spectrum of Avicel, α-Cellulose, D4Mres, and RAC. Hemicellulose is the average of WAX, birch xylan. OSX was excluded in this analysis because it was not considered as pure xylan. Lignin is the average of Alcell, Protobind 100, and Indulin AT. The corresponding average lifetimes were Cellulose 2.16±0.06 ns, Hemicelluloses 2.01 ± 0.25 ns, and Lignin 0.24 ± 0.01 ns.

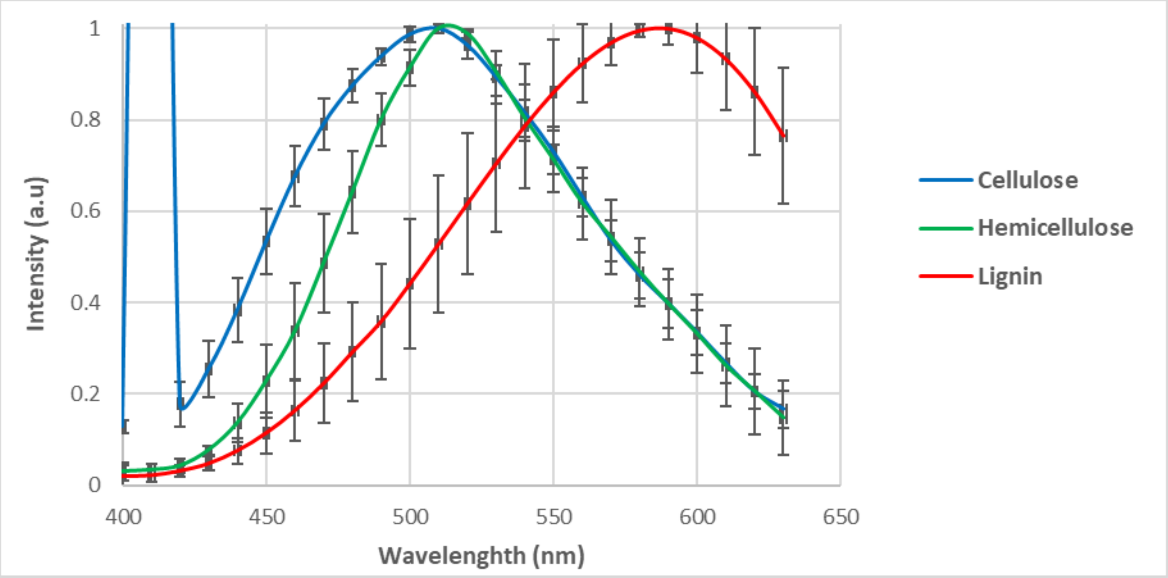

Figure S4. Average of autofluorescence spectra of Cellulose, Hemicellulose, and Lignin.

1.5 Enzyme cocktail autofluorescence

The autofluorescence properties of the enzyme cocktail were measured in water and acidic solution. An appropriate amount of hydrolytic enzyme mixture containing cellulases and hemicellulases (DSM, Delft, Netherlands) was diluted in demineralized water (pH:7) and in 0.2 M acetate buffer (pH:5). In order to detect significant levels of autofluorescence a concentration of enzyme cocktail much higher than the amounts used for the hydrolysis

experiments was used. Results of autofluorescence spectra and lifetime are presented in Figure S5. A small variation in the spectra (Figure S5A) between water and acetate buffer was observed, however the spectral resolution (10 nm) is not enough to measure a shift in the emission peak. Therefore, we conclude that the shift in the emission peak is less than 10 nm. Lifetime was measured at 1629 ± 64 ps in water and 1722 ± 122 ps in the acetate buffer. A different solution and different pH value can affect the properties of fluorescence emission. The effect in the spectra is not detectable with our measurements, but in lifetime an increase in lifetime was measured. The difference is less than 6%, which is rather small, so the pH does not affect significantly the lifetime of the enzyme cocktail.

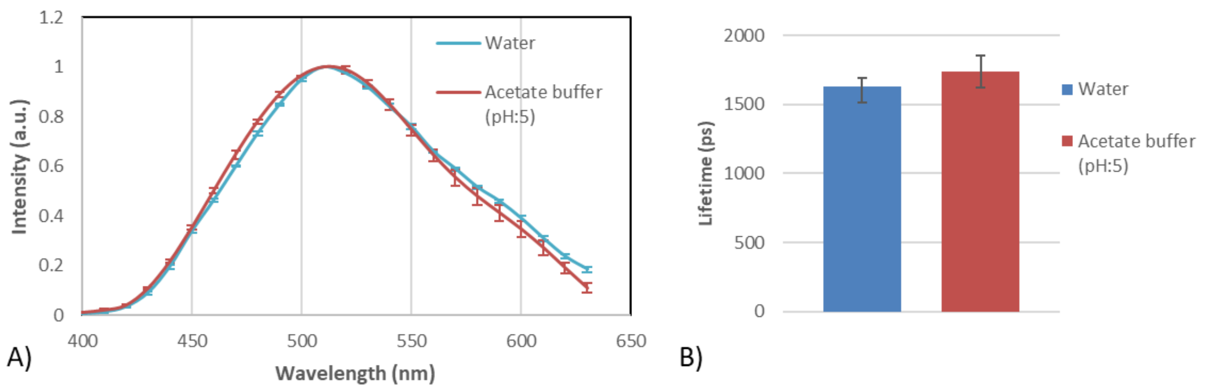

Figure S5. Autofluorescence properties of the enzyme cocktail. **(A)** Autofluorescence spectra in water and acetate buffer. **(B)** Autofluorescence lifetime in water and acetate buffer.

References

(1) Kumar, R.; Wyman, C. E. Physical and Chemical Features of Pretreated Biomass That Influence Macro-/Micro-Accessibility and Biological Processing. In *Aqueous Pretreatment of Plant Biomass for Biological and Chemical Conversion to Fuels and Chemicals*; 2013; pp 281–310.

(2) Kumar, R.; Mago, G.; Balan, V.; Wyman, C. E. Physical and Chemical Characterizations

of Corn Stover and Poplar Solids Resulting from Leading Pretreatment Technologies. *Bioresour. Technol.* **2009**, *100* (17), 3948–3962.

(3) Karimi, K.; Taherzadeh, M. J. A Critical Review of Analytical Methods in Pretreatment of Lignocelluloses: Composition, Imaging, and Crystallinity. *Bioresource Technology*. 2016, pp 1008–1018.

(4) Himmel, M. E.; Park, S.; Johnson, D. K.; Baker, J. O.; Parilla, P. A. Cellulose Crystallinity Index: Measurement Techniques and Their Impact on Interpreting Cellulase Performance. *Biotechnol. Biofuels* **2010**, *3* (1), 10.

(5) Mertz, J.; Moreaux, L. Second-Harmonic Generation by Focused Excitation of Inhomogeneously Distributed Scatterers. *Opt. Commun.* **2001**, *196* (1–6), 325–330.

(6) Matteini, P.; Cicchi, R.; Ratto, F.; Kapsokalyvas, D.; Rossi, F.; de Angelis, M.; Pavone, F. S. S.; Pini, R. Thermal Transitions of Fibrillar Collagen Unveiled by Second-Harmonic Generation Microscopy of Corneal Stroma. *Biophys J* **2012**, *103* (6), 1179–1187.

(7) Brown, Jr., R. M.; Millard, A. C.; Campagnola, P. J. Macromolecular Structure of Cellulose Studied by Second-Harmonic Generation Imaging Microscopy. *Opt. Lett.* **2003**, *28* (22), 2207.

(8) Glas, D.; Paesen, R.; Depuydt, D.; Binnemans, K.; Ameloot, M.; De Vos, D. E.; Ameloot, R. Cellulose Amorphization by Swelling in Ionic Liquid/Water Mixtures: A Combined Macroscopic and Second-Harmonic Microscopy Study. *ChemSusChem* **2015**, *8* (1), 82–86.

(9) Isogai, A.; Atalla, R. H. Amorphous Celluloses Stable in Aqueous Media: Regeneration from SO2–Amine Solvent Systems. *J. Polym. Sci. Part A Polym. Chem.* **1991**, *29* (1), 113–119.

(10) Gong, Y.; Tan, Y.; Mei, J.; Zhang, Y.; Yuan, W.; Zhang, Y.; Sun, J.; Tang, B. Z. Room Temperature Phosphorescence from Natural Products: Crystallization Matters. *Sci. China Chem.* **2013**, *56* (9), 1178–1182.

(11) Olmstead, J. A.; Gray, D. G. Fluorescence Emission from Mechanical Pulp Sheets. *J. Photochem. Photobiol. A Chem.* **1993**, *73* (1), 59–65.

(12) Malinowska, K. H.; Rind, T.; Verdorfer, T.; Gaub, H. E.; Nash, M. A. Quantifying Synergy, Thermostability, and Targeting of Cellulolytic Enzymes and Cellulosomes with Polymerization-Based Amplification. *Anal. Chem.* **2015**, *87* (14), 7133–7140.

(13) van Gool, M. P.; van Muiswinkel, G. C. J.; Hinz, S. W. A.; Schols, H. A.; Sinitsyn, A. P.; Gruppen, H. Two GH10 Endo-Xylanases from Myceliophthora Thermophila C1 with and without Cellulose Binding Module Act Differently towards Soluble and Insoluble Xylans. *Bioresour. Technol.* **2012**, *119*, 123–132.

(14) Radotic, K.; Kalauzi, A.; Djikanovic, D.; Jeremic, M.; Leblanc, R. M.; Cerovic, Z. G. Component Analysis of the Fluorescence Spectra of a Lignin Model Compound. *J Photochem Photobiol B* **2006**, *83* (1), 1–10.

(15) Donaldson, L. A.; Radotic, K. Fluorescence Lifetime Imaging of Lignin Autofluorescence in Normal and Compression Wood. *J Microsc* **2013**, *251* (2), 178–187.
